# Feline heartworm disease and environmental allergens hypersensitivity: is there a link?

**DOI:** 10.1186/s13071-023-05776-3

**Published:** 2023-06-09

**Authors:** Sara N. García-Rodríguez, Noelia Costa-Rodríguez, Jorge I. Matos, Yaiza Falcón-Cordón, Rodrigo Morchón, Elena Carretón, José A. Montoya-Alonso

**Affiliations:** 1 Internal Medicine, Faculty of Veterinary Medicine, Research Institute of Biomedical and Health Sciences (IUIBS), University of Las Palmas de Gran Canaria, Las Palmas de Gran Canaria, Spain; 2 Zoonotic Diseases and One Health Group, Laboratory of Parasitology, Faculty of Pharmacy, University of Salamanca, 37007 Salamanca, Spain

**Keywords:** Heartworm, Allergy, *Dirofilaria immitis*, Cats, Feline, Animal diseases, Pollen, *Dermatophagoides*, *Malassezia*

## Abstract

**Background:**

Cats can be infected by *Dirofilaria immitis*, the causative agent of heartworm disease, characterized by respiratory signs, airway hyperreactivity, remodelling and inflammation. Allergy is a multifactorial pathology, and the role of a number of helminth parasites in the development of allergies in humans and other species has been demonstrated in many studies. The aim of the present study was to verify whether cats seropositive for *D. immitis* present hypersensitivity to some environmental allergens.

**Methods:**

Blood samples were collected from 120 cats and tested for the presence of specific immunoglobulin G antibodies against *D. immitis* and for hypersensitivity to 20 allergens, using commercial allergen test kits.

**Results:**

Of the 120 cats tested, 72 (60.0%) were seropositive for anti-*D. immitis* IgG and 55 (45.8%) showed clinical signs of heartworm disease of a respiratory nature. The results of testing with the allergen kits showed that 50.8% of cats were seropositive for ≥ 1 allergens, with the most common allergens being *Dermatophagoides farinae* (25.8%), *Dermatophagoides pteronyssinus* (20.0%), *Malassezia* (17.5%) and *Ctenocephalides felis* (14.2%). The prevalence of allergies was significantly higher—by almost threefold—in cats seropositive for *D. immitis* (68.1% vs. 25%). There were no significant differences between the prevalence of allergic cats and presence/absence of symptoms, and the results confirmed that symptoms were not a decisive factor for the presence of allergies. The risk for developing allergies was 6.3-fold higher in cats seropositive for *D. immitis* than in cats that were seronegative, confirming that seropositivity for *D. immitis* is a risk factor.

**Conclusions:**

Cats with confirmed heartworm can develop serious respiratory signs, potentially leading to progression to permanent lung injury and predisposing cats to hyperresponsive airway disease. Previous studies have shown that seropositivity for *D. immitis* and *Wolbachia* is related to the presence of bronchoconstriction and bronchospasm in the affected cat. The results support the suspicion that contact with *D. immitis* may be a risk factor for the presence of allergies.

**Graphical abstract:**

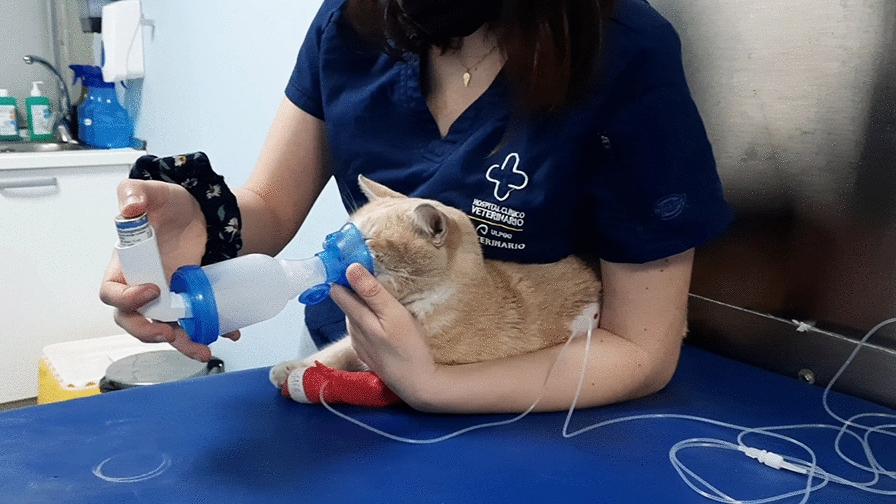

## Background

Cats are susceptible to being infected by *Dirofilaria immitis*, a vector-borne nematode that causes heartworm disease [1, 2]. Feline heartworm disease can present in two forms: (i) when juvenile worms reach the pulmonary arteries, approximately 3–4 months after the initial infection; and (ii) when adult parasites die. Both forms produce an acute inflammatory response in the pulmonary arteries, lung parenchyma and airways, leading to heartworm-associated respiratory disease (HARD) [3, 4].

The clinical signs shown by* D. immitis*-infected cats are mainly respiratory in nature and very similar to those of more frequently diagnosed diseases, such as feline asthma or chronic bronchitis, pathologies with which feline heartworm disease is frequently confused [2]. Moreover, feline asthma or allergic bronchitis causes airway hyperreactivity and remodelling, as well as eosinophilic inflammation, resulting in limitations in airflow; these are also signs similar to those observed in cats with feline heartworm [5, 6]. Dyspnoea, tachypnoea and intermittent coughing are the most common signs of feline heartworm disease, but infected cats may also be asymptomatic or show nonspecific clinical signs, with sudden death sometimes being the only symptom [2–4]. For these reasons, the diagnosis of feline heartworm is complicated and easily overlooked by owners and veterinarians. An additional complication in detecting* D. immitis* infection is that only a low number of adult worms are usually present in the infected cat, necessitating the use of serological techniques (antigen and antibody detection tests) combined with imaging techniques (thoracic radiology and echocardiography) to reach the correct diagnosis.

Allergy is considered to be a multifactorial pathology. Different risk factors for the development of allergies have been described, including a wide set of environmental, infectious (viral agents), genetic and socio-cultural variables. However, other factors may influence the development of allergies, and in many cases the origin of the allergic process is unknown [7].

Studies have shown that some helminth parasites play a role in the development of allergic processes in humans. For example, cases of allergic rhinitis, asthma and atopic dermatitis have been investigated in infections caused by *Ascaris* spp. and *Toxocara* spp. [8–11]. Similar results have been reported for other parasites, such as *Trichuris trichiura*, hookworm and *Schistosoma mansoni* [12, 13]. Moreover, there is evidence that some nematodes, such as *D. immitis*, can increase allergen hypersensitivity in humans [14, 15]. More recently, Montoya-Alonso et al. [7] reported that constant exposure of humans to *D. immitis* may possibly be a risk factor for increased hypersensitivity to allergens.

This relationship has also been studied and confirmed in other vertebrate species (i.e. dogs, mice, pigs) [16–19]. However, any such association has only fleetingly been studied in cats, although with promising results [20, 21].

The usefulness of environmental allergen tests in detecting and managing certain feline respiratory pathologies, such as asthma or allergic bronchitis, has been demonstrated. The basis for performing these tests is that knowledge of the allergens involved can help to develop a long-term treatment plan, adapting the environment to avoid or reduce contact with the allergens [22–24]. For diagnosis, intradermal tests or serum determinations of allergen-specific immunoglobulin E (IgE) for different IgE antibodies have been shown to be useful as only a blood sample is required; furthermore, glucocorticoids and antihistamines are not believed to interfere with this procedure [24].

The aim of this study was to verify whether cats seropositive for *D. immitis,* based on the presence of specific antibodies against the parasite, also present hypersensitivity to some environmental allergens.

## Methods

This study included 120 cats which attended the Veterinary Teaching Hospital of the University of Las Palmas de Gran Canaria (ULPGC), located in a hyperendemic area for heartworm [25]. Cats enrolled in the study were randomly selected from cats which had been brought for consultations related to respiratory symptoms and those which attended scheduled preventive medicine check-ups. All cats were subjected to anamnesis and physical exam. Epidemiological identification data (age, sex, breed) and clinical history data were collected for each animal. Blood samples were taken from the cephalic or jugular vein using 3-ml syringes and 21G needles and collected in serum tubes, followed by centrifugation. The serum was kept at − 20 °C until testing. The inclusion criteria for entry into the study were: (i) age > 6 months; (ii) no previous use of preventative treatment for heartworm; and (iii) no use of medication prior to blood collection.

Samples were tested by indirect enzyme-linked immunosorbent assays (in-house ELISA; Urano Vet®, Barcelona, Spain) for the detection of specific immunoglobulin G (IgG) antibodies against *D. immitis*. Briefly, each well of the ELISA plate was coated with recombinant *D. immitis* antigens (Di33 protein, 0.5 ug/ml). The serum was added to the sample diluent at 1:100 dilution. After a first washing step to remove any molecules that were not bound, the TMB substrate was added, which specifically binds to feline IgG and is labeled with horseradish peroxidase. The absorbances (or optical densities) were read at 450 nm within 5 min after the addition of the stop solution (sulfuric acid). According to the instructions of the manufacturer of the kit, seronegativity was indicated at a cut-off < 1, and seropositivity was indicated at a cut-off of ≥ 1.

The samples were also tested for hypersensitivity to 20 allergens using commercial allergen kits (Polycheck Canis 20 bioassay; Biocheck, Muenster, Germany). The tested allergens were: *Dermatophagoides farinae*, *Dermatophagoides pteronyssinus*, *Lepidoglyphus* spp., *Acarus siro*, *Tyrophagus* spp, *Malassezia*, *Aspergillus*/*Penicillium*/*Alternaria*/*Cladosporodium*, ragweed (Ambrosia) pollen, birch/alder/hazel pollen, platane/willow/poplar pollen, wall pellitory pollen, rye pollen, 6 grass mix, stinging nettle pollen, lambs quarter pollen, plantain pollen, mugwort pollen, sorrel pollen, flea (*Ctenocephalides felis*) and cross-reactive carbohydrate determinant. Each kit contained a plate with 12 cassettes containing specific IgE allergens combined with an enzyme-mediated colour reaction to measure the levels allergen-specific IgE antibodies in the sample. The analysis of the results was performed using the imaging software provided by the manufacturer (Biocheck), which analysed and calculated the image data, indicating the amount of allergen-specific immunoglobulin E (IgE) as relative kiloUnits per litre (kU/l). Samples were considered to be seropositive when the IgE concentration was ≥ 3 kU/l.

Data were analysed using SPSS Base 20.0 software for Windows (SPSS/IBM Corp., Armonk, NY, USA). For categorical variables, frequencies and percentages were shown. For continuous variables, descriptions of the mean, standard deviation (SD), median and interquartile range were displayed. The differences in the parameters between groups for demographic variables were evaluated using Mann–Whitney tests (non-parametric) or Student’s t-test/analysis of variance (ANOVA) (parametric), based on the normality of the variables to be evaluated using the Shapiro-Wilks test. For the comparison of parameters between groups, general linear models (GML) adjusted for age were applied, and the estimated marginal means and their 95% confidence interval (CI) were shown. To detect risk factors of being allergic, we applied a binary logistic regression. The Hosmer–Lemeshow test was used to check the calibration of the model. Stepwise variable selection models were applied to minimize the negative effects of overfitting when working with several predictor variables. All multiple comparisons were adjusted by the Bonferroni correction. All contrasts were accompanied by the effect size estimator to complete the interpretation of the results (Cramer’s V for categorical variables, and Cohen’s D for continuous variables). The significance level used in the analyses was 5% (*α* = 0.05).

All owners were informed of the details of the study and consented to participate. The project was carried out in accordance with the current Spanish and European legislation on animal protection.

## Results

Of the 120 cats included in the study, 58 were male (48.3%) and 62 were female (51.7%). The age of the cats ranged from 6 months to 14 years (mean age ± SD: 3.52 ± 2.9 years), and was higher in those cats which were seropositive to *D. immitis* (*Mann-Whitney; P* < 0.05). Thus, all estimation models were adjusted for age in the group comparisons. The presence of males and females was balanced when all cats were studied and by group comparisons (case/control). The most represented breed was the European Shorthair (88.3%).

Based on the ELISA results, 72 (60.0%) were seropositive to anti-*D. immitis* IgG and 48 (40.0%) were seronegative. Of the 120 cats, 55 (45.8%) showed clinical signs, with all 55 showing respiratory symptoms, and 65 (54.2%) cats were asymptomatic. For study purposes, cats were divided into four subgroups: group A: symptomatic seropositive cats, *n* = 29; group B: asymptomatic seropositive cats, *n* = 43; group C: symptomatic seronegative cats, *n* = 26; and group D: asymptomatic seronegative cats, *n* = 22. There were no significant differences between the presence of symptoms, and the test results of the *D. immitis* ELISA  [GML; F(1.24)= 2.355, *P* = 0.138].

**Fig. 1:**
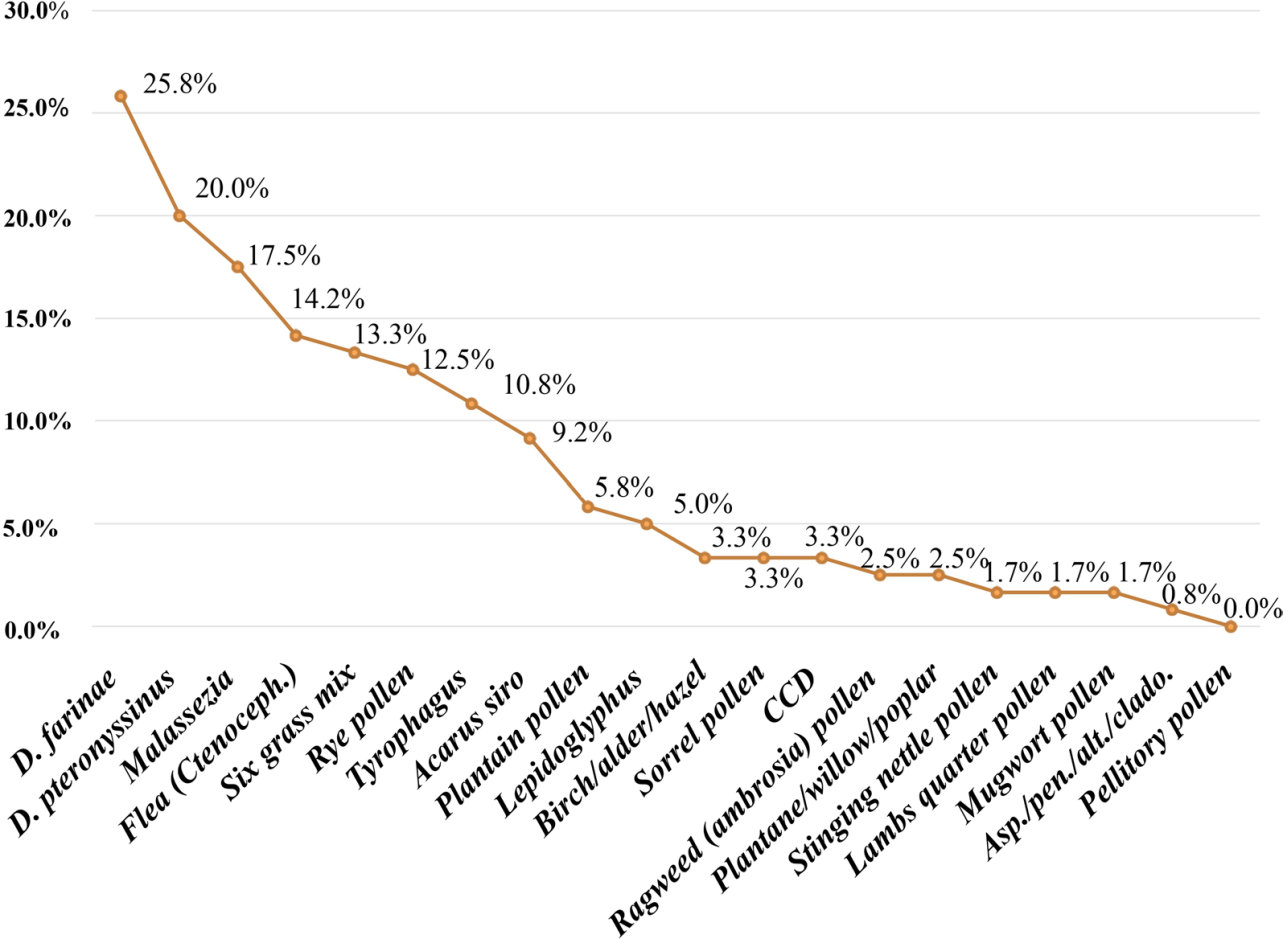
Prevalence obtained for each studied allergy in all the analysed cats.

The results of the allergens kits showed that 50.8% of cats were seropositive to ≥ 1 allergens. The most common allergens were: *Dermatophagoides farinae* (25.8%), *Dermatophagoides pteronyssinus* (20.0%), *Malassezia* (17.5%) and flea (14.2%) (Fig. 1).

While 49.2% of the cats were not seropositive to any allergen, 18.3% showed increased IgE for one allergen, 11.7% for two allergens and 20.7% for > 2 allergens. The mean number of allergies per cat was 1.483 (95% CI 1.012, 1.954), with no significant differences found between seropositive and seronegative cats for *D. immitis* when adjusting for age  [GML; F(1.117)= 2.326, *P* = 0.130]. 

The percentage of cats with allergies was significantly higher in the group of cats seropositive for *D. immitis* [GML; F(1.24)= 20.629, *P* = 0.000; Cramer’s V for effect size = 0.42], with the incidence of allergies being almost threefold higher than that in *D. immitis*-negative cats (68.1% vs 25%). Cats seropositive to *D. immitis* showed higher prevalence of IgE against all of the most common allergens. There were no significant differences in the prevalence of allergic cats and presence/absence of symptoms between *D. immitis*-seropositive and -seronegative cats.

**Fig. 2:**
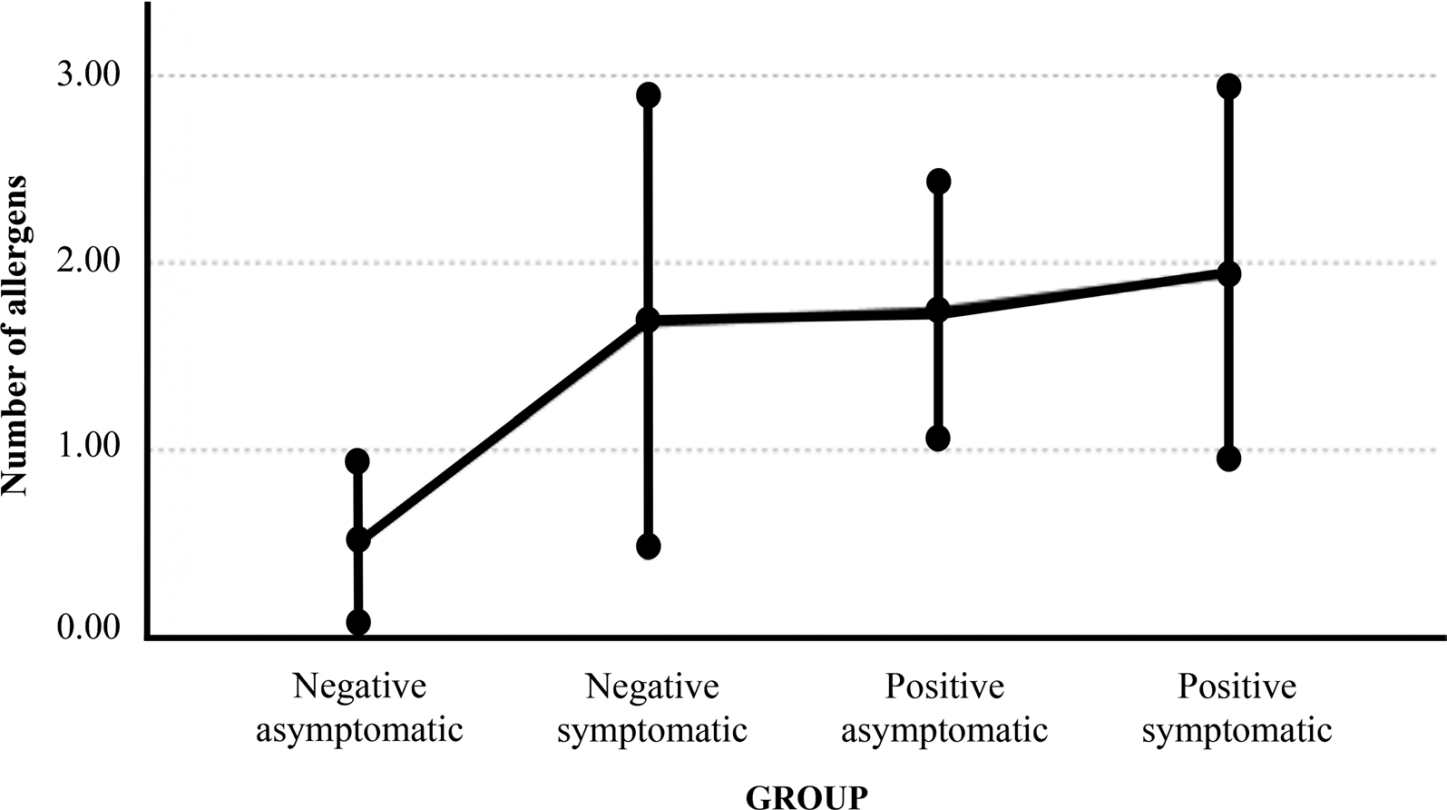
Estimation of the relationship between the number of allergies detected in each cat and the combination of serology to *Dirofilaria immitis*/presence/absence of symptoms. Continuous predictors are fixed at the following value: age = 3.5158 years

The analysis of all four subgroups combined (A + B + C + D) revealed a relationship between the number of allergies per cat and *D. immitis* seropositivity/symptom combination [GML; F(3.115)= 6.038, *P* = 0.001]. For asymptomatic cats, the mean number of allergies per cat (i.e., presence of increased IgE against the studied environmental antigens) was higher in seropositive cats (post-hoc tests with Bonferroni correction: *P* = 0.005, Cohen’s D = 0.48). Similarly, the mean number of allergies was higher in symptomatic seropositive cats than in asymptomatic seronegative cats (*P* = 0.003, Cohen’s D = 0.56). For seronegative cats, the mean number of allergies tended to be higher in symptomatic cats than in asymptomatic ones (*P* < 0.1, Cohen’s D = 0.46) (Fig. 2).

**Fig. 3:**
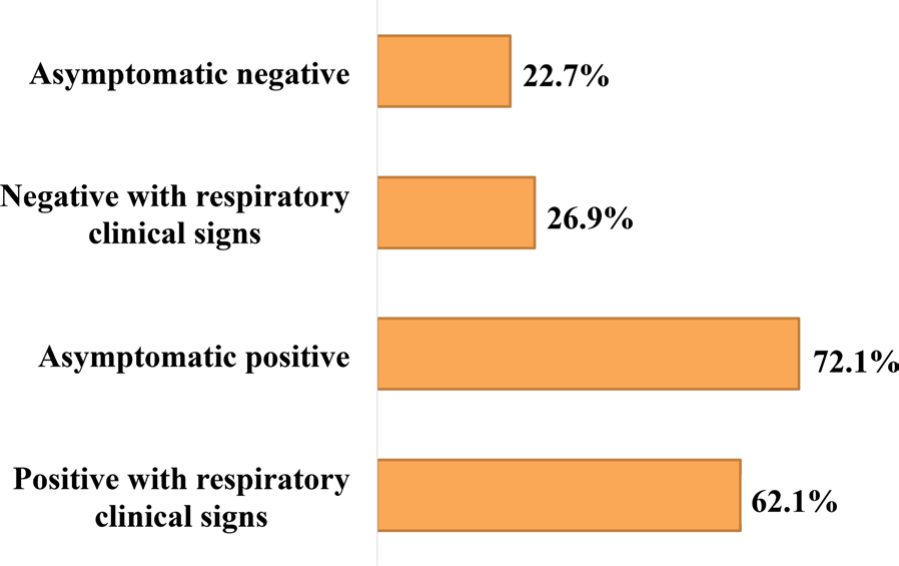
Prevalence of allergic cats by subgroup based on serology to *D. immitis* and presence/absence of clinical signs

When we studied the relationship between all four subgroups combined (A + B + C + D) and the presence of allergies, the results indicated an overall significant relationship [GML; F(3.35)= 7.650, *P* = 0.000, Cramer’s V = 0.430) between the four groups. Post-hoc comparisons adjusted by the Bonferroni test indicated that the incidence of allergies was two- to threefold higher in seropositive cats (both asymptomatic and symptomatic) than in the two seronegative (symptomatic/asymptomatic) subgroups (Fig. 3). The results therefore confirmed that symptoms were not a decisive factor for the presence of allergies.

A logistics regression was applied with a dichotomous dependent variable that coded whether the cat was allergic or not, with “being allergic” the event studied. The multivariate logistics model or logit expressed the probability that a cat may be allergic depending on the different factors considered. Thus, the analysis detected which aspects significantly increased the probability of being allergic. The prognostic factors considered were sex, age, breed, serology to *D. immitis* and respiratory symptoms.

For the final model, the Hosmer and Lemeshow test had a chi-square value of 1.947 and a* P*-value of 0.923. The model had a moderate area under the curve value of 0.707 (95% CI 0.612, 0.801).

The model included only one predictor, namely the *D. immitis* ELISA result; the other factors were not included because Exp(b) was < 1, which indicated a reduction in the probability of allergy. We used the logistic equation to estimate different values of the independent factors. The interpretation of the impact on the risk of the predictors was 6.391 for the result in the *D. immitis* ELISA test, meaning that the risk of being allergic was multiplied by a factor of 6.3 in cats seropositive for *D. immitis* compared to those which were seronegative.

When the variable of the four subgroups was introduced instead of the *D. immitis* result and the symptoms separately, the quality of the model was similar: 0.727 (95% CI 0.636, 0.819), with a chi-square for the Hosmer–Lemeshow test = 15.398 and *P*-value = 0.062. These results showed that being seropositive and asymptomatic multiplied the risk of allergy by 8.7 (compared to being seronegative asymptomatic), being seropositive with symptoms multiplied the risk by 5.6 (compared to being seronegative asymptomatic), while being seronegative with symptoms did not vary the risk of allergy compared to asymptomatic seronegatives. These results confirmed that being seropositive for *D. immitis* was a risk factor for allergy, but that the presence/absence of symptoms was not a risk factor for allergy.

## Discussion

Several studies have shown that various parasitic molecules are involved in the modulation of the immune and allergic response of hosts infected by helminths and have described that parasitic infections can evoke a strong response and high levels of IgE in allergic patients [26, 27].

Previous studies in humans have shown a relationship between helminthic infections and the development of atopy, asthma, or rhinitis, but this relationship is still open to controversy. For example, infections caused by *Toxocara* spp. and *Ascaris lumbricoides* have been reported to cause allergic reactions (such as atopy, rinithis and asthma) in adults and children, with seropositivity to those parasites being a strong risk factor for allergic outcomes in younger generations [11, 27]. In addition, a higher rate of seropositivity for *Toxocara* spp*.* and *A. lumbricoides* was found in children with allergic rhinitis [11, 27, 28]. Also, increased levels of IgE specifically against different filarial species have been found in allergic individuals with asthma or tropical pulmonary eosinophilia [11, 26, 29, 30].

This relationship between parasites and the development of hypersensitivity has also been studied in species other than humans. Fischer et al. [16] showed that dogs infected with *Toxocara canis* had increased IgE levels and allergic reactions to house dust mites (*D. farinae*). Similar results were found in the present study, with *D. farinae*, which is one of the most frequent allergens reported in cats [24, 31, 32], found to be the most prevalent allergen in both the seropositive and seronegative cats. Another survey carried out in mice found an association between *Toxocara cati* infection and asthma [17]. Dawson et al. [19], showed that *Ascaris suum* can cause immediate hypersensitivity in the lungs of pigs when the larvae migrated through the alveolar spaces. In cats, only experimental studies have been carried out to determine the clinicopathological characteristics of asthma after sensitization to ovalbumin [20] and *A. suum* antigens [21], with the results showing persistent airway hyperresponsiveness and histologic alterations.

Regarding *D. immitis*, it has been observed that people who come into contact with this parasite (seropositive to specific IgG) showed the presence of total IgE and specific anti-*D. immitis* IgE more frequently [7], indicating that constant contact with infected vectors may stimulate the development of specific IgE against *D. immitis*, which may be a factor contributing to the development of allergies in inhabitants of hyperendemic regions [7, 15]. In another study, the presence of IgG against *D. immitis* was found to be more frequent in children with asthma [14].

Cats with heartworm show the clinical signs of endarteritis, fibrosis of the vascular intima and hypertrophy of the walls of pulmonary arteries happens, similar to the clinical signs in dogs [2–4]. Moreover, type I cell injury and type II cell hyperplasia occurs in the lungs of cats with heartworm, the consequences of which have not been studied but which could lead to permanent lung injury after worm death and thus predispose cats to hyperresponsive airway disease and other respiratory complications [33]. In this context, previous studies have shown that seropositivity to *D. immitis* and *Wolbachia* surface protein (WSP) is related to the presence of bronchoconstriction and bronchospasm in the cat [6, 34]. Although seropositivity to WSP was not evaluated in the present study, it is not unreasonable to suspect that the contact with *D. immitis* may be a risk factor for the presence of allergies, as suggested by the results of this study.

Analysis of the results showed that the presence of symptoms was not a risk factor for the presence of allergies, nor were there significant differences between the presence/absence of symptoms and *D. immitis* seropositivity. In the cat, it is common to find asymptomatic infections at the time of diagnosis; however, the cats in this study were also at risk of having a fatal outcome and the risk of complications was also present [2–4]. Moreover, the immune system may develop more sensitivity to other allergens after several re-exposures to the parasite, since it is common for allergic animals to present hypersensitivity to various allergens, as observed in the present study [35, 36].

The scientific evidence obtained to date in other parasites and the results obtained from the present study suggest the possibility that the symptoms and pathophysiology of feline heartworm disease may be complicated by allergic pathologies in this species. Consequently, the study of allergen panels could be relevant in infected cats, whether or not they are symptomatic at the time of diagnosis.

There are a number of limitations to this study. The results are not indicative of causation but, rather, correlation, and therefore do not demonstrate that *D. immitis* infection is a causative agent for the development of allergies. Furthermore, cats were studied according to whether or not they were seropositive to IgG against *D. immitis*. The presence of antibodies indicates exposure, but not necessarily current infection. However, those cats with the presence of specific IgG are considered to be in contact with the parasite and are therefore at risk of infection. Also, to exclude other diseases (i.e. infectious diseases [parasites and others], cardiac diseases, neoplasia), all cats were mainly subjected to anamnesis, physical exam, thoracic X-ray, echocardiographic exam and blood tests. To detect IgE against several common allergens, only a diagnostic test was performed, with no additional confirmatory examinations. Moreover, only 20 allergens were evaluated, so the possible involvement of other allergens was not studied. Furthermore, the sensitivity and specificity of these tests were not specified by the manufacturer, so these are also important limiting factors to take into account. Finally, all cats were from an area where heartworm is hyperendemic, but the variability of the allergic antigenic load to which the study cats may have been exposed is unknown. Nevertheless, the selected cats came from very similar climatic areas. Further studies to determine the possible relationship between *D. immitis* infection and the development of allergies need to be carried out.

## Conclusions

The results of the present study support the suspicion that cats which have been in contact with *D. immitis* are more likely to exhibit an immune response against environmental allergens. We also found that being seropositive was a risk factor for the development of hypersensitivity in cats. Given the limitations of the study, the results should be interpreted with caution, although they do provide interesting information which should encourage future studies focused on determining whether there is a causal relationship between the development/exacerbation of allergies and *D. immitis* infection.

## Data Availability

The datasets used and/or analysed during the current study are available from the corresponding author on reasonable request.

## Abbreviations

HARD: Heartworm-associated respiratory disease
WSP: *Wolbachia* surface protein
